# Deliberately making miskates: Behavioural consistency under win maximization and loss maximization conditions

**DOI:** 10.1038/s41539-023-00206-6

**Published:** 2023-12-06

**Authors:** Yajing Zhang, Thi Kim Truc Huynh, Benjamin James Dyson

**Affiliations:** 1 University of Alberta, Edmonton, Canada; 2 Toronto Metropolitan University, Toronto, Canada

**Keywords:** Human behaviour, Decision making

## Abstract

We argue that the feedback traditionally used to indicate negative outcomes causes future detrimental performance because of the default goal of *win maximization*. In gaming paradigms where participants intentionally performed as well (*win maximization*) and as poorly (*loss maximization*) as possible, we showed a double dissociation where actions following wins were more consistent during *win maximization*, but actions following losses were more consistent during *loss maximization*. This broader distinction between *goal-congruent* and *goal-incongruent* feedback suggests that individuals are able to flexibly redefine their definition of ‘success’, and provide a reconsideration of the way we think about ‘losing’.

How did you react to the error in our title “*Deliberately making miskates*”? If you assumed that this was unintentional, then your reaction was probably an unfavourably negative one: we have been careless in our proofreading. However, in learning that the error was intentional, your reaction loses some of its negative connotations. This example clearly demonstrates that there is an interaction between the properties of a stimulus and the goal behind stimulus production^1,2^. Orthographically, the printed word “miskates” remains a spelling error, independently of whether the spelling was unintentional or intentional. However, the negativity associated with this error can be reconciled with the knowledge of intentionally incorrect spelling. In this paper, we empirically investigate the interaction between the positive or negative nature of a stimulus, and, whether the production of that stimulus is incongruent or congruent with the goal set by the individual.

A dominant goal in most organismic behaviour is *win maximization*, typically defined as gaining as many/much as one can^3–5^. Both experimental and everyday tasks are similarly framed in terms of doing one’s best: a memory test might require participants to try to remember in as much detail as possible^6^, a perceptual test might insist that performance is both as fast and as accurate as possible^7^, and, an individual visiting a casino hopes to leave with as much money as possible. Therefore, with behaviour oriented towards the goal of *win maximization*, it should not be surprising that when participants receive feedback that a memory detail is inaccurate, a perceptual error has been made, or money is lost, the delivery of this information can have negative consequences^8–10^. Furthermore, *Operant Conditioning*^11,12^ dictates that there are very clear consequences for future actions on the basis of current outcomes: organisms will tend to repeat actions after a win (*win-stay)* but change actions after a loss (*lose-shift)*. Although *lose-shift* behaviour is sub-optimal, it remains more predictable than *win-stay*^11^ and also becomes more likely as the cognitive load of the task increases^12^. By these accounts, we would do well to avoid losses^13^.

However, a reimagining of the consequences of putatively negative feedback is possible by considering the role of goal-directed learning. When your goal is *win-maximization*, on any trial that you are informed that you lost there is a discrepancy between your goal state and your current state (consistency theory; see refs. ^14,15^). This discrepancy we will refer to as *goal-incongruent* feedback. By this logic however, a second example of discrepancy presumably arises under the unique case when the goal of losing is violated by the experience of winning. To be specific, if *win maximization* is the goal, then each individual win represents *goal-congruent* feedback, whereas each individual loss represents *goal-incongruent* feedback. Conversely, if *loss maximization* is the goal, each individual loss represents *goal-congruent* feedback, whereas each individual win represents *goal-incongruen*t feedback.

Therefore, much of the data accrued on the negative impact of losing may simply be due to the implicit (or explicit) goal of *win maximization*. If reactions to wins and losses are essentially arbitrary and individuals are able to flexibly redefine their definition of success, we should also see wins disrupting behaviour when the organism aims to lose. We carried out four experiments in which participants competed against computerized opponents under conditions of *win-maximization* and *loss-maximization*. In all experiments, different forms of computerized strategy were designed such that optimized participant behavior was either aligned or misaligned with the operant conditioning principles of *win-stay* and *lose-shift*. In Experiment 1, participants played *Matching Pennies* with the goal of *win-maximization*, where ‘wins’ represented *goal-congruent* and ‘losses’ represented *goal-incongruent* feedback. Our data showed that behavioral consistency was disrupted when goal and feedback were incongruent: specifically, performance was worse following losses relative to wins when the goal was *win maximization*. In Experiment 2, participants played the same game with the explicit goal of *loss-maximization*. Again, consistency was disrupted when the goal and feedback were incongruent but the behavioural observation was reversed: performance was worse following wins relative to losses when the goal was *loss maximization*. In Experiment 3, we confirmed the between-participant observations of Experiments 1 and 2 by running a within-participants design. In Experiment 4, we extended these findings to a different game (*Dice Dual*) involving 6 rather than 2 responses. Across Experiments 1–4, we also found no evidence that behaviour was significantly optimized when participant strategy was aligned with operant conditioning principles. Thus, our data demonstrate a double dissociation where behaviour following wins was more consistent during *win maximization*, but behaviour following losses was more consistent during *loss maximization*.

## Results

### Measuring behavioural consistency

Across the experimental series, we test the idea that individuals are able to flexibly redefine their definition of success by measuring behavioural consistency when playing a game intentionally both as well as possible (i.e., *win maximization*) and as badly as possible (i.e., *loss maximization*). During *win maximization*, putatively positive feedback (‘*win’*) represents *goal-congruency*. Conversely, negative feedback (‘*lose’*) represents *goal-congruency* during *loss maximization*. If receiving negative feedback is inherently detrimental, then performance should be universally less consistent following losses relative to wins. If, instead, behavioural disruption is determined by *goal incongruency*, performance should be less consistent following *loss* trials within the context of *win maximization* but also less consistent following *win* trials within the context of *loss maximization*.

Across Experiments 1–4, the strategy required for exploitation was determined by both the nature of opponency (*repetition, alternation*) and maximization goal (*win, loss*). As summarized in Table 1, in the case of *win maximization* for the *repetition* opponent, goal-consistent performance was guaranteed via the expression of traditional *win-stay* and *lose-shift* mechanisms: participants repeat their previous response following a win but change their previous response following a loss. However, *win maximization* for the *alternation* opponent could only be achieved by expressing the opposing rules: *win-shift* and *lose-stay*. These assignments were reversed when the goal was *loss maximization*: *win-shift* and *lose-stay* were required to lose against the *repetition* opponent, and, *win-stay* and *lose-shift* were required to reliably lose against the *alternation* opponent. As a result of this design, we were also able to assess the ‘default’ nature of actions traditionally associated with wins and losses as dictated by operant conditioning (*win-stay / lose-shift*; see^16,17^). If there is hardwired precedence for *win-stay* and *lose-shift*, then performance should be more consistent when strategy aligns with these principles (specifically, *win maximization + repetition* opponent, and, *loss maximization + alternation* opponent). However, if these outcome-action associations are arbitrary, consistency in expressing *win-shift* should be similar to *win-stay*, as should consistency in expressing *lose-stay* relative to *lose-shift*.

**Table 1:** Opponency structure and optimal strategies in Experiments 1–4 where [H] = Heads [T] = Tails [O] = Odd number [E] = Even number.

Opponent	Optimal Strategy		
		Win Maximization (Exp. 1, 3)	Loss Maximization (Exp. 2, 3)
*Unexploitable*			
[HHHHH] + [TTTTT] (random)	x 9	*n/a*	*n/a*
*Exploitable via repetition*			
[HHHHH] + [TTTTT] (random)	x 3	*n/a*	*n/a*
[HHHHHHHHHH]	x 3	*Win-Stay / Lose-Shift*	*Win-Shift / Lose-Stay*
[TTTTTTTTTT]	x 3	*Win-Stay / Lose-Shift*	*Win-Shift / Lose-Stay*
*Exploitable via alternation*			
[HHHHH] + [TTTTT] (random)	x 3	*n/a*	*n/a*
[HTHTHTHTHT]	x 3	*Win-Shift / Lose-Stay*	*Win-Stay / Lose-Shift*
[THTHTHTHTH]	x 3	*Win-Shift / Lose-Stay*	*Win-Stay / Lose-Shift*
		Win Maximization (Exp. 4)	Loss Maximization (Exp. 4)
*Unexploitable*			
[OOOOO] + [EEEEE] (random)	x 9	*n/a*	*n/a*
*Exploitable via repetition*			
[OOOOO] + [EEEEE] (random)	x 3	*n/a*	*n/a*
[OOOOOOOOOO]	x 3	*Win-Stay / Lose-Shift*	*Win-Shift / Lose-Stay*
[EEEEEEEEEE]	x 3	*Win-Stay / Lose-Shift*	*Win-Shift / Lose-Stay*
*Exploitable via alternation*			
[OOOOO] + [EEEEE] (random)	x 3	*n/a*	*n/a*
[OEOEOEOEOE]	x 3	*Win-Shift / Lose-Stay*	*Win-Stay / Lose-Shift*
[EOEOEOEOEO]	x 3	*Win-Shift / Lose-Stay*	*Win-Stay / Lose-Shift*

### Experiment 1 (win maximization)

**Fig. 1:**
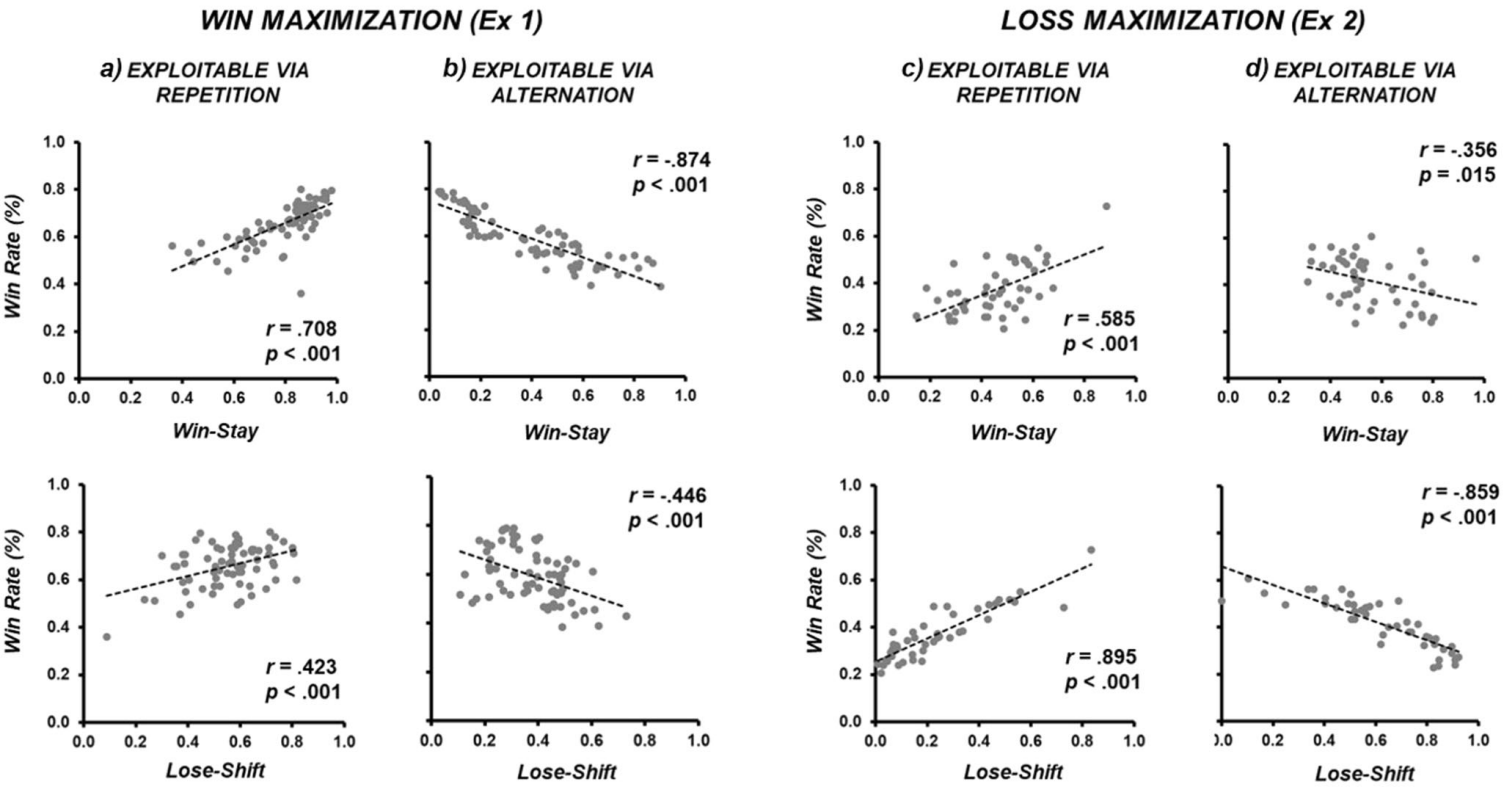
Scatterplots of *win-stay* and *loss-shift* behaviour as a function of individual win rate, and the nature of goals and opponency. A double dissociation is observed between Experiments 1 and 2. **a**, **b** describe results in Experiment 1: the degree of behavioural consistency is significantly greater following a winning rather than losing outcome when the goal is *win maximization*; (**c**, **d**) describe results from Experiment 2: the degree of behavioural consistency is significantly greater following a losing rather than winning outcome when the goal is *loss maximization*. In both Experiments 1 and 2, this is irrespective of whether the action is consistent or inconsistent with the fundamental reinforcement learning principles of *win-stay* and *lose-shift*.

Win rates (proportion of a participant’s winning trials over all trials), the degree of *win-stay* (a proportion of *win-stay* trials over the total number of winning trials), and the degree of *lose-shift* (a proportion of *lose-shift* trials over the total number of losing trials) are calculated across Experiments 1–4 (see Table 2). During *win maximization* against an opponent *exploitable via repetition* (Experiment 1; Fig. 1a), both the degree of *win-stay* (*r* = 0.708, *p* < 0.001) and *lose-shift* (*r* = 0.423, *p* < 0.001) were positively correlated with win rate. By comparing the absolute values of the abovementioned correlation coefficients (i.e., *r* = 0.708 and *r* = 0.423) using a *z*-test (two-tailed *z* test; see^18,19^), we found that stay actions following *wins* had a significantly stronger correlation than shift actions following *losses* (*z* = 2.347, *p* = 0.019). Therefore, performance was more consistent following wins relative to losses in the context of *win maximization*.

**Table 2:** Descriptive statistics of win rates and degrees of *win-stay* and *lose-shift* across Experiments 1-4 Standard error in parenthesis.

	Win Maximization			Loss Maximization		
	Win Rate	*Win-stay*	*Lose-shift*	Win Rate	*Win-stay*	*Lose-shift*
Experiment 1						
*Exploitable via repetition*	0.657 (.013)	0.800 (.018)	0.557(.020)		n/a	
*Exploitable via alternation*	0.601 (.015)	0.370 (.032)	0.381 (.016)			
Experiment 2						
*Exploitable via repetition*		n/a		0.369 (.018)	0.452 (.029)	0.228 (.032)
*Exploitable via alternation*				0.420 (.018)	0.569 (.026)	0.609 (.036)
Experiment 3						
*Exploitable via repetition*	0.655 (.013)	0.787 (.019)	0.557 (.015)	0.394 (.017)	0.535 (.023)	0.239 (.023)
*Exploitable via alternation*	0.603 (.014)	0.342 (.029)	0.431 (.017)	0.420 (.014)	0.615 (.020)	0.576 (.029)
Experiment 4						
*Exploitable via repetition*	0.607 (.014)	0.724 (.022)	0.524 (.019)	0.416 (.014)	0.521 (.021)	0.311 (.025)
*Exploitable via alternation*	0.583 (.013)	0.383 (.027)	0.427 (.019)	0.426 (.014)	0.288 (.018)	0.588 (.031)

With the same goal of *win maximization* against an opponent *exploitable via alternation* (Fig. 1b), both the degree of *win-stay* (*r* = -0.874, *p* < 0.001) and *lose-shift* (*r* = -0.446, *p* < 0.001) were negatively correlated with win rate. This is consistent with the requirement to *win-shift* and *lose*-*stay* (see Method). Actions following *wins* once again had a significantly stronger correlation than actions following *losses* (*z* = 5.019, *p* < 0.001). Therefore, performance was more consistent following wins relative to losses in the context of *win maximization*. Thus, the data from Experiment 1 are clear that the requirement to deploy either *stay* or *shift* behaviour as a function of opponent was more consistent following *wins* relative to *losses*, when the goal of the task is *win maximization*.

### Experiment 2 (loss maximization)

During *loss maximization* against an opponent *exploitable via repetition* (Experiment 2; Fig. 1c), both *win-stay* (*r* = 0.585, *p* < 0.001) and *lose-shift* (*r* = 0.895, *p* < 0.001) behaviour were again positively correlated with win rate. In contrast to Experiment 1, actions following *losses* now had a significantly stronger correlation than actions following *wins* (*z* = 3.895, *p* < 0.001). Therefore, performance was more consistent following losses relative to wins in the context of *loss maximization*. Against an opponent *exploitable via alternation* (Fig. 1d), both the degree of *win-stay* (*r* = -0.356, *p* = 0.015) and *lose-shift* (*r* = -0.859, *p* < 0.001) were negatively correlated with win rate. This is consistent with the requirement to *win-shift* and *lose-stay* (see Method). Actions following *losses* had a significantly stronger correlation than actions following *wins* (*z* = 4.057, *p* < 0.001), showing that performance was more consistent following losses relative to wins in the context of *loss maximization*. Collectively, Experiments 1 and 2 represent a double dissociation. When the goal of the task was *win maximization*, the deployment of either *stay* or *shift* behaviour was more consistent following wins relative to losses, but when the goal of the task was *loss maximization*, the deployment of either *stay* or *shift* behaviour was more consistent following *losses* relative to *wins*.

### Experiments 3 and 4 (*win* and *loss maximization*)

**Fig. 2:**
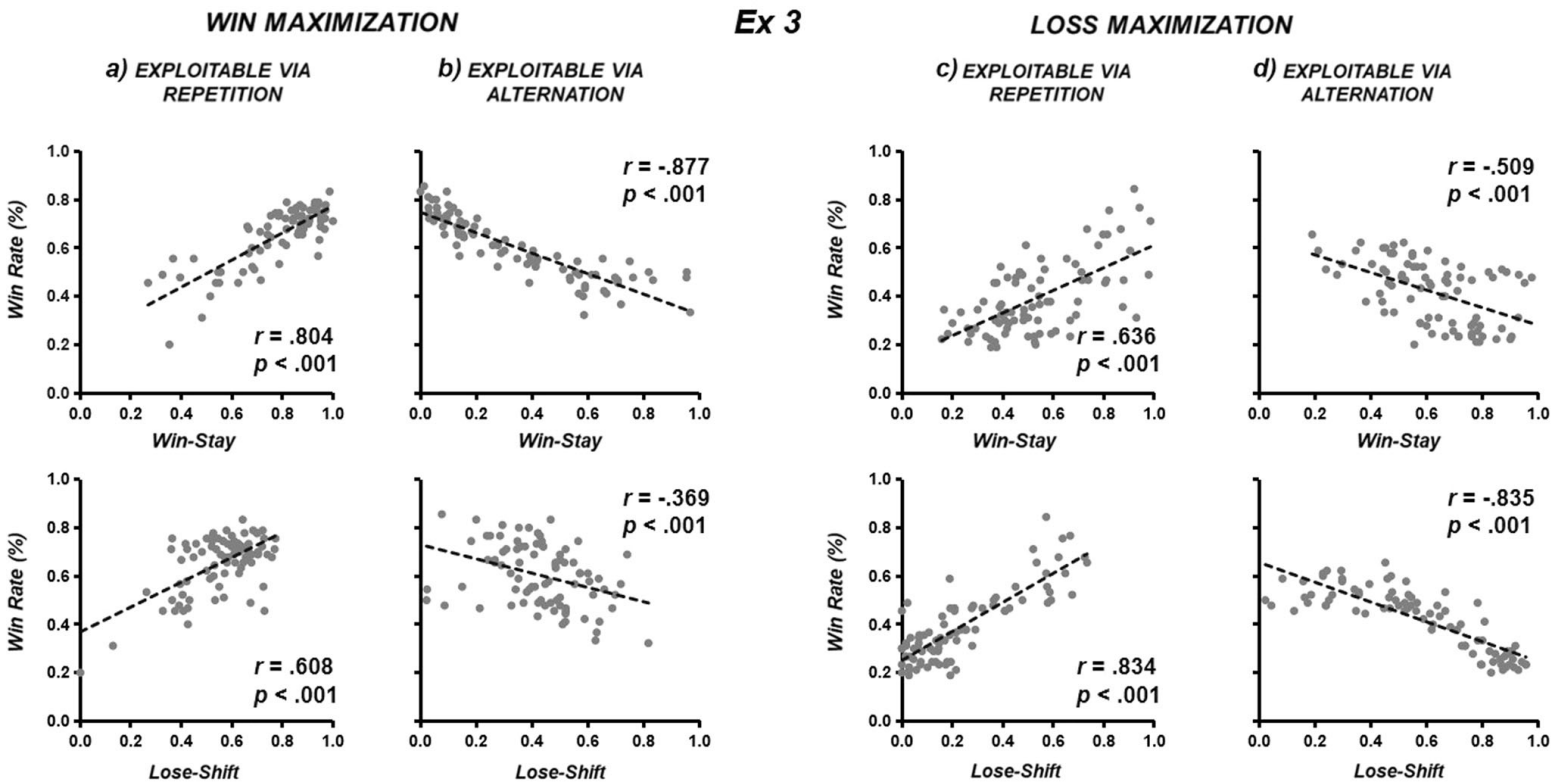
The double dissociation between Experiments 1 and 2 replicates in the within-participants design of Experiment 3. **a**, **b** Both *win-stay* and *win-shift* are more consistent in the context of *win maximization*, however, (**c**, **d**) both *lose-stay* and *lose-shift* are more consistent in the context of *loss maximization* (right four panels).

**Fig. 3:**
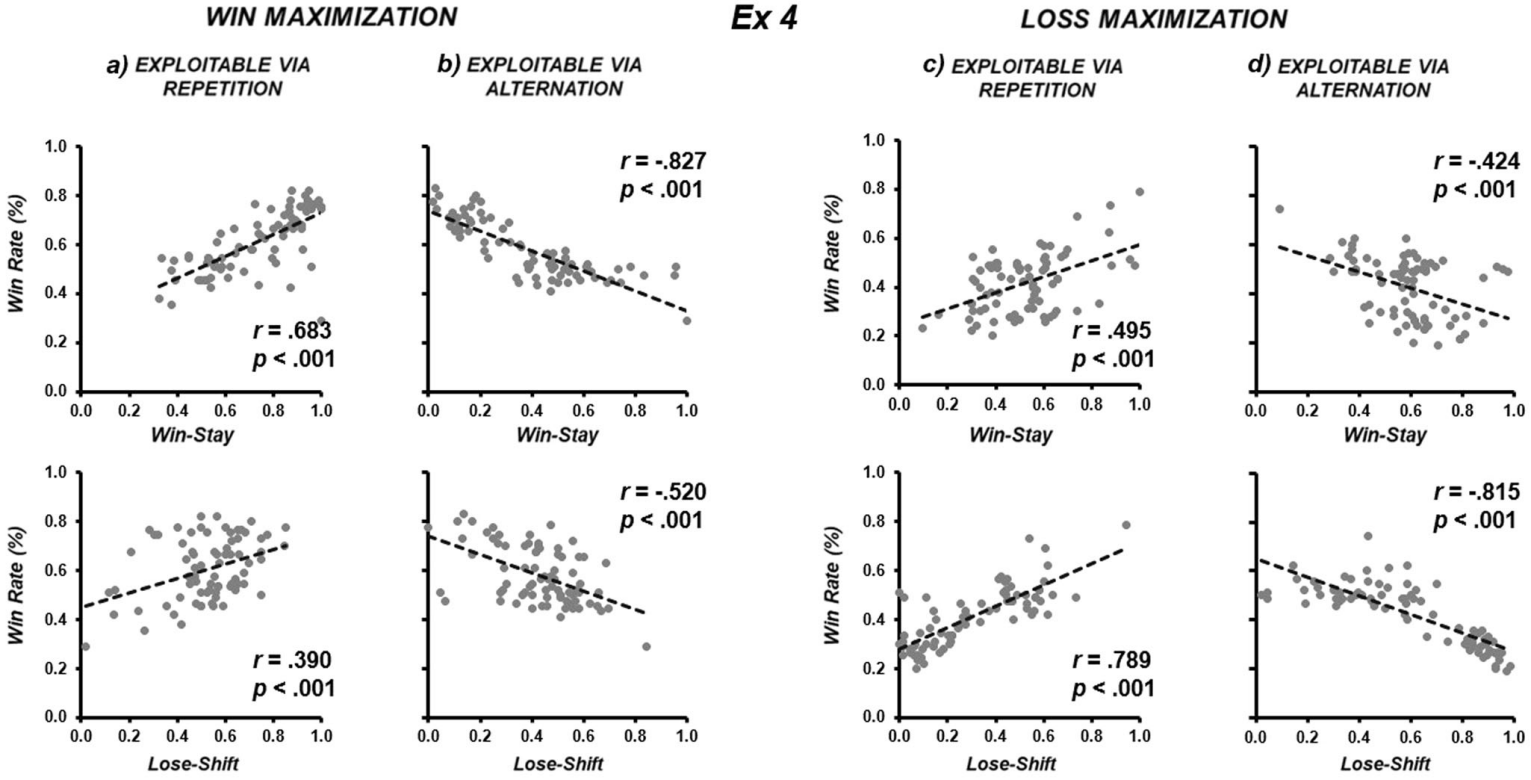
The double dissociation between Experiments 1 and 2 replicates in the within-participants design of Experiment 4. **a**, **b** Both *win-stay* and *win-shift* are more consistent in the context of *win maximization*, however (**c**, **d**) both *lose-stay* and *lose-shift* are more consistent in the context of *loss maximization* (right four panels).

This same double dissociation observed in the within-participants design of Experiment 3 (again using the 2-response game *Matching Pennies;* see Fig. 2) was similarly replicated in the within-participants design of Experiment 4 (using the 6-response game *Dice Dual*; see Fig. 3). That is, the behaviour was more consistent both following wins relative to losses in the context of *win maximization* but also more consistent following losses relative to wins in the context of *loss maximization*.

In the context of *win maximization* against an opponent *exploitable via repetition*, the ability to enact *win-stay* behaviour was more consistent than the ability to enact *lose-shift* behaviour (Experiment 3: *z* = 2.744, *p* = 0.006; Fig. 2a; Experiment 4: *z* = 2.370, *p* = 0.018; Fig. 3a). In the context of *win maximization* against opponent *exploitable via alternation*, the negative correlation between win rate and *win-stay* behaviour was more consistent than *lose-shift* behaviour (Experiment 3: *z* = 6.047, *p* < 0.001; Fig. 2b; Experiment 4: *z* = 3.6676, *p* < 0.001; Fig. 3b). In the context of *loss maximization* against opponent *exploitable via repetition*, the ability to enact *lose-shift* behaviour was more consistent than the ability to enact *win-stay* behaviour (Experiment 3: *z* = 2.250, *p* = 0.024; Fig. 2c; Experiment 4: *z* = 3.1041, *p* = 0.002; Fig. 3c). In the context of *loss maximization* against opponent *exploitable via alternation*, the negative correlation between win rate and *lose-shift* was more consistent than *win-stay* behaviour (Experiment 3: *z* = 4.060, *p* < 0.001; Fig. 2d; Experiment 4: *z* = 3.9443, *p* < 0.001; Fig. 3d).

### Comparing win-stay / lose-shift against win-shift / lose-stay

Furthermore, we observed no evidence that *win-stay/lose-shift* were privileged forms of outcome-action association. In the context of *win maximization* in Experiment 1, participants were significantly less consistent in their ability to deploy *win-stay* behaviour (during *repetition*) than *win-shift* behaviour (during *alternation*) to intentionally increase win rate (*r* = 0.708 vs [abs] *r* = 0.874; *z* = -2.935, *p* = 0.003; two top left panels of Fig. 1). In the context of *loss maximization* (Experiment 2), participants were no less consistent in their ability to deploy *lose-stay* behaviour (during *repetition*) than *lose-shift* behaviour (during *alternation*) to intentionally decrease win rate ([abs] *r* = 0.895 vs. *r* = 0.859; *z* = 1.283, *p* = 0.199; two bottom right panels of Fig. 1). In Experiment 3, *win-stay* behaviour (during *repetition*) was numerically less consistent than *win-shift* behaviour (during *alternation*) during *win maximization* (*r* = 0.804 vs [abs] *r* = 0.877; *z* = -1.601, *p* = 0.109; two top left panels of Fig. 2). No difference was shown between *lose-shift* behaviour (during *alternation*) and *lose-stay* behaviour (during *repetition*) during *loss maximization* ([abs] *r* = 0.835 vs. *r* = 0.834; *z* = 1.086, *p* = 0.278; two bottom right panels of Fig. 2). In Experiment 4, *win-stay* behaviour (during *repetition*) was significantly less consistent than *win-shift* behaviour (during *alternation*) during *win maximization* (*r* = 0.683 vs [abs] *r* = 0.827; *z* = -1.9632, *p* = 0.0496; two top left panels of Fig. 3). No difference was shown between *lose-shift* behaviour (during *alternation*) and *lose-stay* behaviour (during *repetition*) during *loss maximization* ([abs] *r* = 0.815 vs. *r* = 0.789; *z* = 0.4173, *p* = 0.677; two bottom right panels of Fig. 3).

As an additional test of whether *win-stay / lose-shift* represented privileged forms of outcome-action associations, we also examined reaction times to see whether the speed to initiate these specific responses was faster than their reversed counterparts: *win-shift / lose-stay*. However, across Experiments 1–4, we found no significant interaction between outcome and action, such that *win-stay* and *lose-shift* timings were not significantly faster than *win-shift* and *lose-stay* timings (see Supplement Materials). Therefore, increased behavioural consistency following wins when the goal-state was winning (and following losses when the goal-state was losing) was independent of whether the individual is required to repeat (*stay*) or change (*shift*) their response to maintain maximization.

## Discussion

There are two major implications of this work. First, our data show that people are flexible in defining ‘success’ specifically via the malleable interpretation of putatively positive (*win*) and negative (*lose*) forms of feedback. We suggest that the traditional disruption generated by losses is the result of incongruity with the goal (implicit or explicit) of *win maximization*^20,21^. In support of this idea, we similarly showed disruption generated by wins due to an equivalent incongruity between feedback and goal in the context of *loss maximization*. This shows the top-down control we have over the interpretation of feedback, and the clear interaction between the properties of a stimulus and the goal behind stimulus production^1,2^. Our observations that behaviour is similarly disrupted by losing in the context of pursuing wins, and, winning in the context of pursuing losses are also consistent with the framework of goal-directed learning^22,23^. In the current studies, when the goal was to maximize wins (Experiments 1, 3 and 4), there was little conflict between what people were asked to do and the goal of *win maximization* implicit in most daily interactions. Much more unusual was the request to adjust one’s goal towards *loss maximization* (Experiments 2, 3 and 4), and our interest was in whether the same behavioural inconsistency would be demonstrated when putatively positive feedback (*win*) became the source of goal incongruency. Although our data suggest flexibility in goal-directed learning, one caveat is the degree to which the sensory properties of the visually-presented words ‘win’ and ‘lose’ constitute intrinsically positive and negative signals. One reason for the flexibility we observed may be because the use of points system represents abstracted rather than concrete forms of feedback. Therefore, it will be of interest to see whether behaviour consistency remains isomorphic between *win maximization* and *loss maximization* conditions when more tangible rewards and punishments are associated.

Second, our data show a remarkable ease with which we can switch out of putatively fundamental operant conditional outcome-action associations represented by *win-stay* and *lose-shift*^24^. Our fluid adaptation to counter reinforcement-learning strategies such as *win-shift* is consistent with data from nectarivorous birds and other organisms who also flexibly adapt to environments that have high depletion rates^25,26^. However, humans may be unique in our ability to simulate the sabotaging of our own performance in pursuit of the goal of *loss maximization* demonstrated here. For examples within popular culture, see the board game Go For Broke^27^, wherein players must *lose* $1 million dollars, and, the UK entertainer Les Dawson who delighted audiences with deliberately terrible piano playing. Moreover, we have shown that both of these findings are observed in both binary (Experiments 1–3) and non-binary (Experiment 4) decision-making spaces, thereby lending credence to the generalizability of these findings.

In conclusion, the historical emphasis on *win-stay* and *lose-shift* mechanisms, and the presumed disruption of performance as a result of losses, are due to the often unspoken goal of *win maximization* within empirical sciences. We have shown that incongruent performance generated by the experience of losing is simply the result of a mismatch between an expected goal state and the current observed state. The same mismatch *and the same consequences of that mismatch* are produced when the intention is to lose but instead the organism wins. The flexibility with which individuals can define ‘success’ and specifically the interpretation of negative feedback is consistent with other observations such as deliberately seeking loss as in the principle of ‘fun failure’^28,29^ or demonstrating cognitive proficiency via the act of intentional self-sabotage. This highlights the importance of subjective interpretation in decision-making, and may help to explain the individual differences associated with emotional reactions to feedback. Finally, we may wish to re-evaluate the extent to which our understanding of human and animal behaviour sciences has been limited by our implicit focus on *win maximization*.

## Method

Data from convenience samples of 71 (Mean = 19.77, SD = 3.12, 33 female), 46 (Mean = 19.2, SD = 1.67, 23 female), 84 (Mean = 19.47, SD = 3.25, 40 female), and 77 (Mean = 20.84, SD = 6.12, 38 female) participants were analyzed from the student population at the University of Alberta for Experiments 1, 2, 3, and 4, respectively. These sample sizes exceed the 30–40 participants previously analysed for zero-sum games in our lab (see refs. ^13,30^), and, the minimum sample size of 29 participants for each experiment calculated using G*Power^31^ with large treatment effect (i.e., correlation ρ H1 = 0.5), alpha value at 0.05, and power level at 0.8. All participants were provided with written informed consent. They gave their informed consent for inclusion before they participated in the study. They completed the study for course credit and the on-line protocol was approved by the University of Alberta Research Ethics Committee (Pro00102699 and Pro00112365). Paradigms were controlled by Presentation 20.2 (build 07.25.18) and delivered remotely after participants downloaded Presentation Package Player. Two exclusion criteria were implemented: (1) procedural: where a participant stopped and re-started the paradigm thereby completing at least one condition more than once, and (2) behavioural: where a participant selected the same item 100% of the time throughout at least one condition. 8 (Experiment 1), 8 (Experiment 2), 22 (Experiment 3), and 6 (Experiment 4) participants were excluded from analyses according to the procedural criterion, and a further 4 (Experiment 1), 3 (Experiment 2), 8 (Experiment 3), and 2 (Experiment 4) participants were excluded from analyses according to the behavioural criterion. Data patterns across Experiments 1–4 remain consistent when participants excluded due to the behavioural criteria were added to the sample.

### Experiments 1–3

For Experiments 1–3, participants played 540 rounds of *Matching Pennies* consisting of 6 conditions each containing 90 trials. Each trial, both participant and computerized opponent selected either Heads or Tails. Participants won the trial if coin sides mismatched and lost the trial if coin sides matched. The 6 counterbalanced conditions consisted of the presence or absence of a cumulative score, crossed with three different kinds of opponency (*unexploitable, exploitable via repetition, exploitable via alternation*; see Table 1). Our factor pertaining to cumulative score yielded no notable effects, so we collapse across score manipulation.

The 90 trials per condition were subdivided into 9 groups of 10 trials each, with groups randomized within conditions. For the *unexploitable* opponent, each group consisted of 5 Heads and 5 Tails response which were further randomized within each group (e.g., TTHTHHHTHT). For the *exploitable via repetition* opponent, 3 blocks were identical to the *unexploitable* opponent, 3 blocks consisted of 10 presentations of Heads in a row (e.g., HHHHHHHHHH), and, 3 blocks consisted of 10 presentations of Tails in a row (e.g., TTTTTTTTTT). For the *exploitable via alternation* opponent, 3 blocks were once again identical to the *unexploitable* opponent, 3 blocks consisted of 10 coin alternations beginning with a Head (e.g., HTHTHTHTHT), and, 3 blocks consisted of 10 coin alternations beginning with a Tail (e.g., THTHTHTHTH). Consequently, all opponents played an equal percentage (50%) of Heads and Tails within each condition. The unexploitable conditions served as a filler task between two exploitable conditions. Because there were no optimal strategies to be employed in unexploitable condition to maximize either wins or losses, we did not include the results from the condition in our analyses and focus exclusively on exploitable opponency.

At each trial, participants would press one of the two buttons corresponding to Heads [K] or Tails [L] prompted by a fixation cross. Both participant and opponent selections were shown on the left and right side of screen, respectively, for 1000 ms. Selections were removed during a 500 ms pause, followed by either “WIN (+1)” or “LOSE (-1)” in green and red font, respectively, for 1000 ms. Scores were updated and the fixation cross returned.

In Experiment 1, the goal was *win maximization*. Before *win maximization* conditions, participants were instructed to “Just try to do as well as you can!” In Experiment 2, the goal was *loss maximization*. Before *loss maximization* conditions, participants were instructed to “Just try to do as BADLY as you can! Try to get the most negative score.” In Experiment 3, both *win maximization* (cf., Experiment 1) and *loss maximization* (cf., Experiment 2) goals were completed in a within-participants design across 6 counterbalanced conditions, without the cumulative score manipulation.

### Experiment 4

To examine whether the data exhibited for the binary-response game *Matching Pennies* generalized to non-binary paradigms, participants played 540 rounds of the lab-designed game *Dice Dual* in Experiment 4. Here, participants and computerized opponents chose one number from the six sides of a die using 6 linearly organized keys. Participants won the trial if the sum of the two sides was even and lost the trial if the sum was odd. Thus, *Dice Dual* was structurally isomorphic to *Matching Pennies* (the actual odd or even die side was irrelevant) but had surface differences with respect to the number of responses available. For the *unexploitable* opponent, each group randomized 5 odd numbers and 5 even numbers (e.g., 1563224516). For the *exploitable via repetition* opponent, 3 blocks were identical to the *unexploitable* opponent, 3 blocks consisted of 10 presentations of odd numbers in a row (e.g., 5331311551), and, 3 blocks consisted of 10 presentations of even numbers in a row (e.g., 4622442466). For the *exploitable via alternation* opponent, 3 blocks were once again identical to the *unexploitable* opponent, 3 blocks consisted of 10 number alternations beginning with an odd number (e.g., 5416321256), and, 3 blocks consisted of 10 number alternations beginning with an even number (e.g., 2152361456). Consequently, all opponent types played an equal percentage (50%) of odd and even numbers within each condition. In Experiment 4, both *win maximization* and *loss maximization* goals were completed in a counterbalanced, within-participants design (as per Experiment 3).

### Reporting summary

Further information on research design is available in the Nature Research Reporting Summary linked to this article.

### Supplementary information

Supplementary materials

Reporting summary

Supplementary dataset 1

Supplementary dataset 2

## Data Availability

All data is available in the Supplement Materials.
