# Supplementary material for: Deliberately making miskates: Behavioural consistency under win maximization and loss maximization conditions
Source: NPJ Sci Learn. 2023 Dec 6;8:55. doi: 10.1038/s41539-023-00206-6 (PMC10700323; doi:10.1038/s41539-023-00206-6)
Supplement: Supplementary file 1 — Supplementary materials [file 41539_2023_206_MOESM1_ESM.pdf]

Experiment 1 – Matching Pennies win maximization

(n = 70)		
	Mean	*Standard error*
Win-stay	423	40
Win-shift	420	37
Lose-stay	350	23
Lose-shift	359	34

Supplementary Table 1 Descriptive statistics for reaction time in Experiment 1

	df	*F*	*MSE*	*p*	η_p^2
Outcome (O)	1,69	**7.477**	**42244**	**.008**	**.098**
Action (A)	1,69	.018	30170	.894	.000
O x A	1,69	.073	37579	.788	.001

Supplementary Table 2 Inferential statistics for reaction time in Experiment 1. Two-way repeated measures ANOVA results. Outcome (O): 2 levels (Win / Lose); Action (A): 2 levels (Stay / Switch)

Note. Significant effects in bold font

Experiment 2 – Matching Pennies loss maximization

(n = 42)

	Mean	*Standard error*
Win-stay	402	42
Win-shift	386	37
Lose-stay	433	46
Lose-shift	392	35

Supplementary Table 3 Descriptive statistics for reaction time in Experiment 2

	df	*F*	*MSE*	*p*	η_p^2
Outcome (O)	1,41	1.003	14076	.323	.024
Action (A)	1,41	1.748	19107	.193	.041
O x A	1,41	.396	17597	.533	.010

Supplementary Table 4 Inferential statistics for reaction time in Experiment 2. Two-way repeated measures ANOVA results. Outcome (O): 2 levels (Win / Lose); Action (A): 2 levels (Stay / Switch)

Note. Significant effects in bold font

Experiment 3 – Matching Pennies collapsed across win & loss maximization

(n = 75)		
	Mean	*Standard error*
Win-stay	517	57
Win-shift	536	66
Lose-stay	493	52
Lose-shift	475	47

Supplementary Table 5 Descriptive statistics for reaction time in Experiment 3

	df	*F*	*MSE*	*p*	η_p^2
Outcome (O)	1,74	3.729	36736	.057	.048
Action (A)	1,74	<.001	41438	.980	.000
O x A	1,74	.857	30420	.358	.011

Supplementary Table 6 Inferential statistics for reaction time in Experiment 3. Two-way repeated measures ANOVA results. Outcome (O): 2 levels (Win / Lose); Action (A): 2 levels (Stay / Switch)

Note. Significant effects in bold font

Experiment 4 – Dice Dual collapsed across win & loss maximization

(n = 74)		
	Mean	*Standard error*
Win-stay	402	31
Win-shift	426	38
Lose-stay	412	39
Lose-shift	454	51

Supplementary Table 7 Descriptive statistics for reaction time in Experiment 4

	df	*F*	*MSE*	*p*	η_p^2
Outcome (O)	1,73	1.191	22221	.279	.016
Action (A)	1,73	**5.763**	**13876**	**.019**	**.073**
O x A	1,73	.490	11842	.486	.007

Supplementary Table 8 Inferential statistics for reaction time in Experiment 4. Two-way repeated measures ANOVA results. Outcome (O): 2 levels (Win / Lose); Action (A): 2 levels (Stay / Switch)

Note. Significant effects in bold font
